# Field experiments of different fracturing designs in tight conglomerate oil reservoirs

**DOI:** 10.1038/s41598-022-07162-y

**Published:** 2022-02-25

**Authors:** Daiyan Zhang, Shiying Ma, Jing Zhang, Yue Zhu, Bin Wang, Jian Zhu, Xibin Fan, Hu Yang, Tianbo Liang

**Affiliations:** 1 CNPC Xinjiang Oilfield Company, Karamay, 834000 China; 2 State Key Laboratory of Oil and Gas Resources and Prospecting, China University of Petroleum at Beijing, Beijing, 102249 China

**Keywords:** Energy science and technology, Fossil fuels, Petrol

## Abstract

Mahu oilfield is currently the largest tight conglomerate reservoir in the world, where Ma-131 and Ma-18 plays are the first two commercially developed reservoirs. In order to reduce the cost and explore the best fracturing parameters, field experiments have been conducted in these two plays since 2017. Types of proppant and fracturing fluid, the slickwater ratio, and the fracture spacing are mainly changed for comparison, and fracturing effects are evaluated to establish a reference for developing neighboring plays in the Mahu oilfield. This paper summarizes the fracturing parameters and production histories of 74 wells in Ma-131 and Ma-18 plays during four years of field operations. Firstly, results indicate that silica sands perform similar to ceramics in the Ma-131 play where the reservoir depth is smaller than 3300 m; however, in the Ma-18 play where the reservoir is deeper than 3500 m, increasing the sand volume by 1.1–1.2 times still cannot reach the production in wells using ceramics. Secondly, when the fracture spacing is reduced, both oil production and water flowback become even smaller in wells using sands than those using ceramics; this is due to the increase of closure pressure and decrease of fluid volume per cluster respectively. Thirdly, when the crosslinked guar is replaced by the slickwater, no obvious change in oil production is noticed even though the volume of fracturing fluid is almost doubled; limited lengths of propped fractures due to the poor proppant-carrying ability of slickwater likely offset the production enhancement from the decrease of formation damage by slickwater. This paper summarizes learnings from the field experiments during the four-year development of the Mahu oilfield, and help guide the optimization of hydraulic fracturing parameters for future wells.

## Introduction

Hydraulic fracturing is necessary to develop low-permeability reservoirs, where a large volume of fracturing fluid is pumped into the horizontal wellbore to generate fractures within each fracturing stage. After the pad is pumped, proppants are added into the fracturing fluid and pumped into the created fractures to prevent them from closure. Currently, ceramic proppants and silica sands are two major types of proppants applied in the field. Ceramic proppants have higher strengths and lower crushing rates comparing to silica sands; laboratory measurements have indicated that the conductivity of ceramic proppants can be 5 to 10 times of silica sands at low to moderate closure stresses, and ceramic sands may loss the conductivity at a high closure stress of 60 MPa^1,2^. However, ceramic proppants have a larger density, which is typically 1–1.5 times of silica sands. Since the settling rate of a proppant in the fracturing fluid changes linearly with its density according to the Stocks equation, the silica sand can migrate further than the ceramic proppant under the same pumping condition, and thus generating longer propped fractures^3^. Similar observations have been published in studies on proppant settling and migration in rough fractures or non-Newtonian fluids^4–7^. To reduce the settling rate of ceramic proppants, the lightweight proppant has been developed, whose density can be reduced to about 75% of silica sands, and the conductivity is enhanced by 40% compared with silica sands^8^. However, its high price limits its field application in China. Besides, laboratory studies have also shown that the fracture roughness and tortuosity can also affect the proppant settling and transportation^4,9,10^, but they are hard to quantify based on laboratory results, especially for heterogeneous reservoirs like tight conglomerates in Mahu. Therefore, it is necessary to compare the performance of different types of proppants through field experiments, so as to explore the conditions when silica sands can partially or completely replace the ceramic proppants.

During hydraulic fracturing, proppants are delivered to the created fracturing by the fracturing fluid, whose viscosity also affects the settling rate of proppants. Laboratory measurements have shown that proppants are almost suspended in the crosslinked gel without settling, which is far slower than the settling rate predicted by the Stocks equation based on the Newtonian fluid assumption^4,11^. The crosslinked gel increases the fluid viscosity and thus can increase the fracture height^12–14^. However, the crosslinked gel cannot completely degrade and its residues can adsorb on the rock surface, thus inhibiting the hydrocarbon flow from the reservoir rock to the created fractures; meanwhile, the residues can also plug the propped fractures and reduce the fracture conductivity^15–17^. Laboratory measurements have shown that the conductivity reduction due to gel residues can be as large as 50–60%^18,19^. Slickwater is made of water and the friction reducer, whose concentration is typically about 0.1%^20^. The friction reducer can reduce the friction of fracturing fluid by more than 70%, thus allowing a significantly larger pumping rate than the crosslinked gel; meanwhile, this large pump rate can slow down proppant settling in the slickwater and carry proppants further towards the fracture tips. Therefore, the crosslinked gel and the slickwater have their advantages and disadvantages, where the former has a better proppant-carrying ability and larger conductivity, and the latter has a lower price and causes less formation damage after fracturing. Since this is difficult to comprehensively evaluate the performance of two types of fracturing fluids, it is necessary to conduct field experiments to explore the best fracturing design for the future development of Mahu tight conglomerate reservoirs.

When the hydraulic fracture propagates, adjacent reservoir rocks are compressed, which can increase the minimal horizontal stress and change the direction of fracture propagation. This is called the “stress shadow effect”, and can cause the non-uniform propagation of fractures within one fracturing stage^21–23^. Recently, along with the downhole characterization techniques including the tracers, downhole camera, the distributed temperature sensing (DTS) and the distributed acoustic sensing (DAS), it has been found 80% production may come from only 20% clusters^24–27^. Observations from reservoir core samples from the Hydraulic Fracturing Test Site (HFTS) have also indicated that hydraulic fractures form the “fracture swarm”, where proppants unevenly distributes among them; when silica sands are used, thick fractures can be observed, but 80% propped fractures are less than 2 mm at a distance of 40–60 m away from the wellbore^28–30^. For low permeability reservoirs, decreasing the fracture spacing can potentially increase the estimated ultimate recovery rate (EUR), but this can intensify the stress shadow effect and reduce the conductivity of created fractures. Therefore, the impact of fracture spacing on chosen proppants is also necessary to be revealed in field experiments.

Since the year of 2017, 74 wells have been chosen in Ma-131 and Ma-18 plays for field experiments, where different types of proppants and fracturing fluids, slickwater ratios, and fracture spacings are tested and compared. This paper summarizes and analyzes observations from field experiments during these four years, and the learnings can help guide the optimization of hydraulic fracturing parameters for future wells in the Mahu reservoirs.

## Target reservoir and field experiments designs

Mahu oilfield is currently the largest tight conglomerate reservoir in the world, with an estimated reserve of over 1.2 billion tons of crude oil. The Ma-131 play has been discovered in 2012, whose pay zone is in the Baikouquan formation (T_1_*b*) at depths around 3100–3300 m. After a few early exploration wells reach commercial oil flow rates through small-scale fracturing, horizontal wells with large-scale fracturing are planned as field experiments between 2015 and 2017. The Ma-18 play is in the southwest of the Ma-131 play, whose pay zone is also T_1_*b* but with greater depths of around 3400–3600 m. Permeability of the reservoir rock in Ma-131 and Ma-18 plays ranges from 0.05 to 8 mD with an average of around 1 mD.

Deeper reservoirs have larger closure stresses and thus can significantly affect the conductivity of propped fractures. Therefore, silica sands and ceramic proppants are mainly compared in both plays to explore the possibility and conditions of substituting ceramics with sands to save operation costs. To compensate the conductivity loss when using silica sands, a larger proppant volume is designed for sand wells during the field experiments (1.1 times the proppant volume of the ceramic well). During the propagation of hydraulic fractures, reservoir rock adjacent to the opening fractures is compressed, which increases the minimal horizontal stress perpendicular to fracture faces. This effect is named as “stress shadow effect”, which not only results in a nonuniform propagation of fractures within one fracturing stage, but also increases the closure stress that can aggravate the proppant crushing and further decrease fracture conductivity. Therefore, proppant replacement is also evaluated at a decreasing fracture spacing in the field experiments.

Crosslinked guar has a good proppant-carrying ability and a low leak-off coefficient; therefore, it is suitable for fracturing vertical wells where tall fractures are needed. However, degradation residues of the crosslinked guar can cover the fracture face and even block the propped fracture, thus inhibiting the hydrocarbon flow from the reservoir rock to the wellbore. Along with the progress of fracturing techniques, slickwater has been widely used as the fracturing fluid, especially in reservoirs with permeabilities of sub-millidarcies to microdarcies where long and complex fractures are needed. In the field experiments, the reverse hybrid fracturing method is applied: the crosslinked guar is first pumped to initiate the fractures; then, the slickwater is pumped with slugs of proppants to make fractures grow, polish the perforations, and prevent the early screen-out; in the end, the crosslinked guar is pumped again with a high concentration of proppant to support hydraulic fracture open. The slickwater ratio is gradually increased from 2015 to 2018 during the field experiments to reduce the potential formation damage and save operation costs. Since proppants have a larger settling rate in the slickwater than in the crosslinked guar, a larger pumping rate is used during the slickwater fracturing. This results in an increase of the total volume of fracturing fluid and can potentially increase the stimulation volume of the reservoir, which is also evaluated in the field experiments.

## Results

### Laboratory measurements of proppant conductivity and settling rate

**Figure 1:**
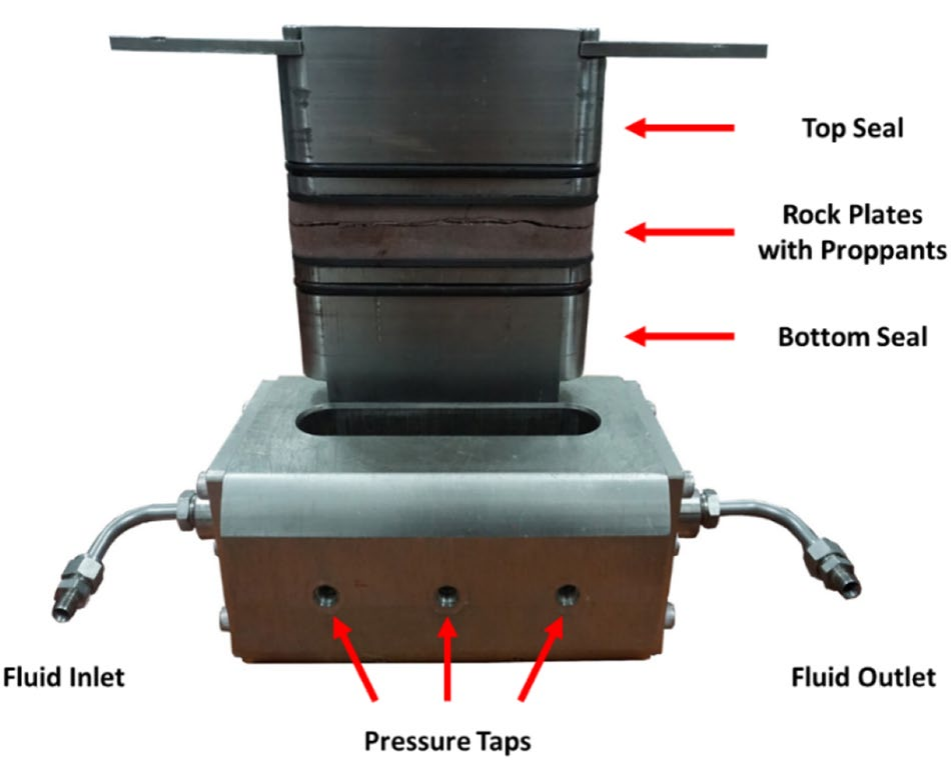
Testing cell for conductivity measurements.

Before different proppants are applied in the field experiments, their conductivities and settling rates are measured in the lab. A length of reservoir core with a diameter of 10 cm is drilled from the Baikouquan formation (T_1_*b*), which is then cut into smaller plates as required by the API standard for measuring the long-term proppant conductivity. Each rock plate has a length of 17.7 cm, a width of 3.8 cm, and a thickness of 1.8 cm. During the conductivity measurement, 8 kg/m^2^ of proppants (silica sands or ceramics) is filled between a pair of rock plates, and then loaded into a testing cell as shown in Fig. 1. After the cell is put in a pressing machine for applying the closure pressure, 2%KCl is continuously flowed through the cell. At each closure pressure, the pressure drop across the cell after 50 h of KCl flooding is recorded for calculating the conductivity using the Darcy’s law; this is repeated for closure pressures increasing from 27.6 to 69.0 MPa.

**Figure 2:**
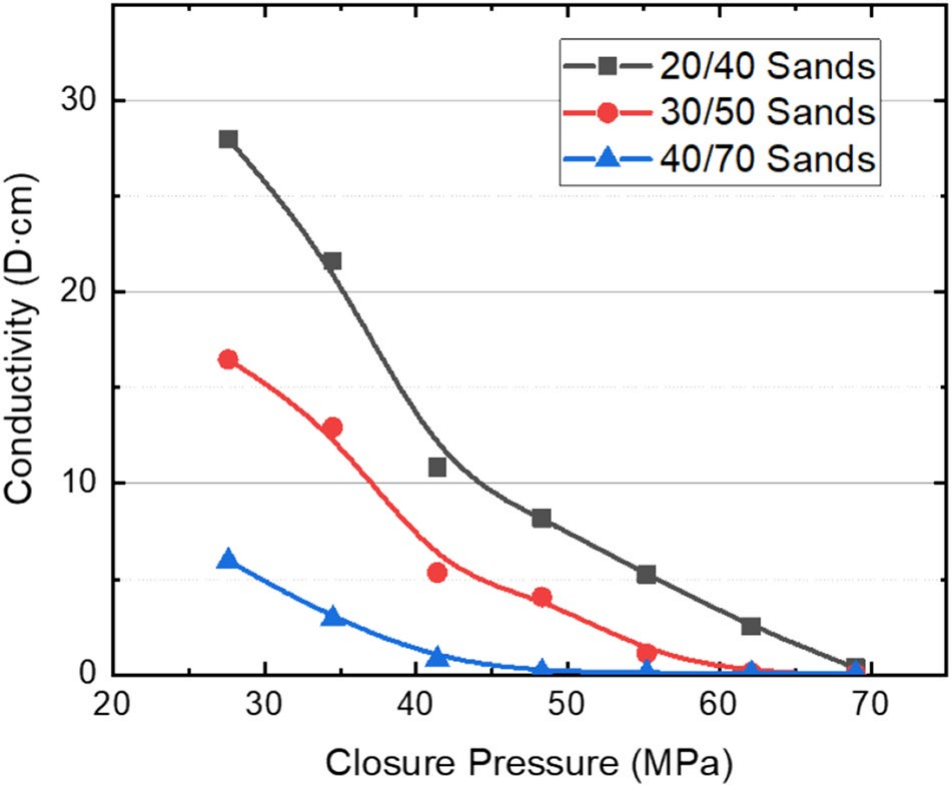
Fracture conductivity of silica sands using reservoir rock plates with a concentration of 8 kg/m^2^.

**Figure 3:**
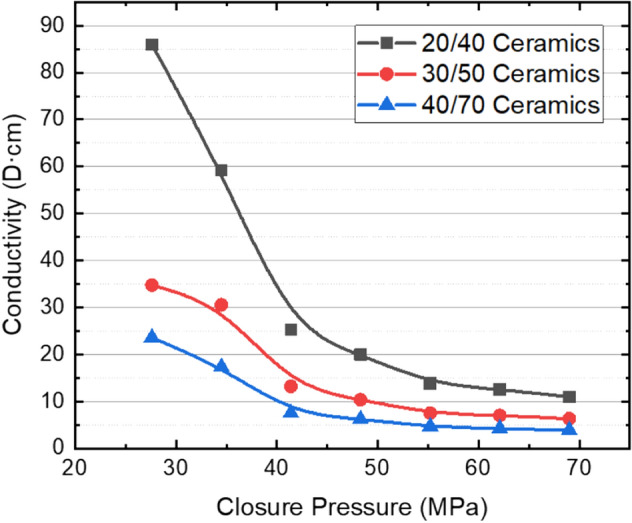
Fracture conductivity of ceramic proppants using reservoir rock plates with a concentration of 8 kg/m^2^.

Figures 2 and 3 show the changes of conductivity of silica sands and ceramic proppants with different meshes and closure pressures in reservoir rock plates. Both figures show the proppant conductivity decreases with the proppant diameter and closure pressure as known already. For 20/40 mesh silica sands, the conductivity decreases from 27.9 to 0.4 D cm when the closure pressure increases from 27.6 to 69.0 MPa. When the sand size decreases to 30/50 mesh, the propped fracture almost loses the conductivity at 62 MPa; when the sand size further decreases to 40/70 mesh, the closure pressure when the propped fracture almost loses the conductivity decreases to 48 MPa. Ceramic proppants have higher strength, better roundness and sphericity comparing to silica sands; therefore, they have larger conductivity and decreases less steeply than silica sands. At relatively low closure pressures, the conductivity of ceramic proppants is about 3 times of silica sands at the same mesh. Even at a closure pressure of 69 MPa, the conductivity of ceramic proppants at 40/70 mesh can be as large as 3.5 D cm, and thus being able to support hydraulic fractures open at a deep reservoir.

**Figure 4:**
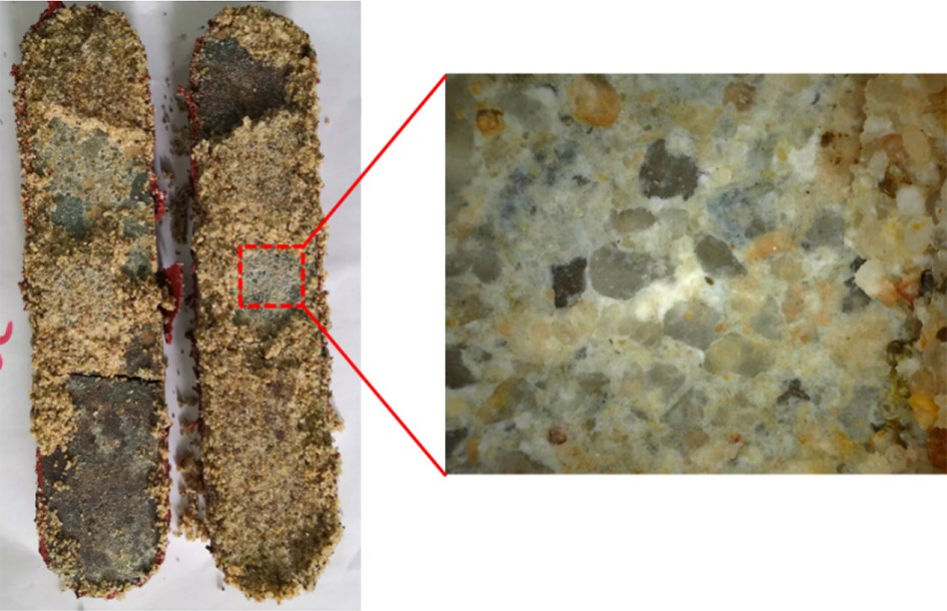
Crushing and embedment of silica sands in reservoir rock plates after a long-term fracture conductivity test.

**Figure 5:**
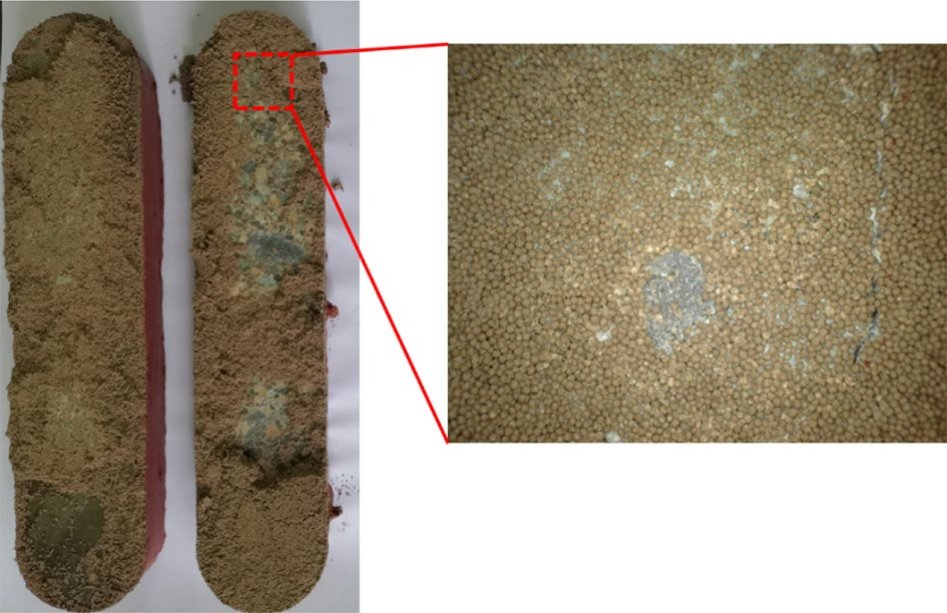
Crushing and embedment of ceramic proppants in reservoir rock plates after a long-term fracture conductivity test.

After the conductivity measurement, rock plates are taken out of the testing cell for observation. Figure 4 shows the open plates containing silica sands after the long-term conductivity test. The crushing of silica sands is almost ubiquitous, which creates a large number of fine particles plugging the propped fracture and reduces the fracture conductivity. However, the embedment of sands into the rock plates is not observed, and it is not the reason causing the reduction of fracture conductivity shown in Fig. 2. Figure 5 shows the open plates containing ceramic proppants after the long-term conductivity test. Compared to the case with silica sands, the crushing of ceramic proppants can be noticed but less common, which allows the propped fracture to maintain a moderation conductivity when the pore pressure decreases and the closure pressure increases during the production. Meanwhile, after ceramic proppants are cleaned from the rock surface, small pits can be observed due to the embedment of proppants. However, when comparing with the conductivity data measured from the API method, one can find that crushing is still the main reason causing the conductivity reduction of the ceramic-propped fracture, although it is less significant than that in the sand-propped fracture.

**Figure 6:**
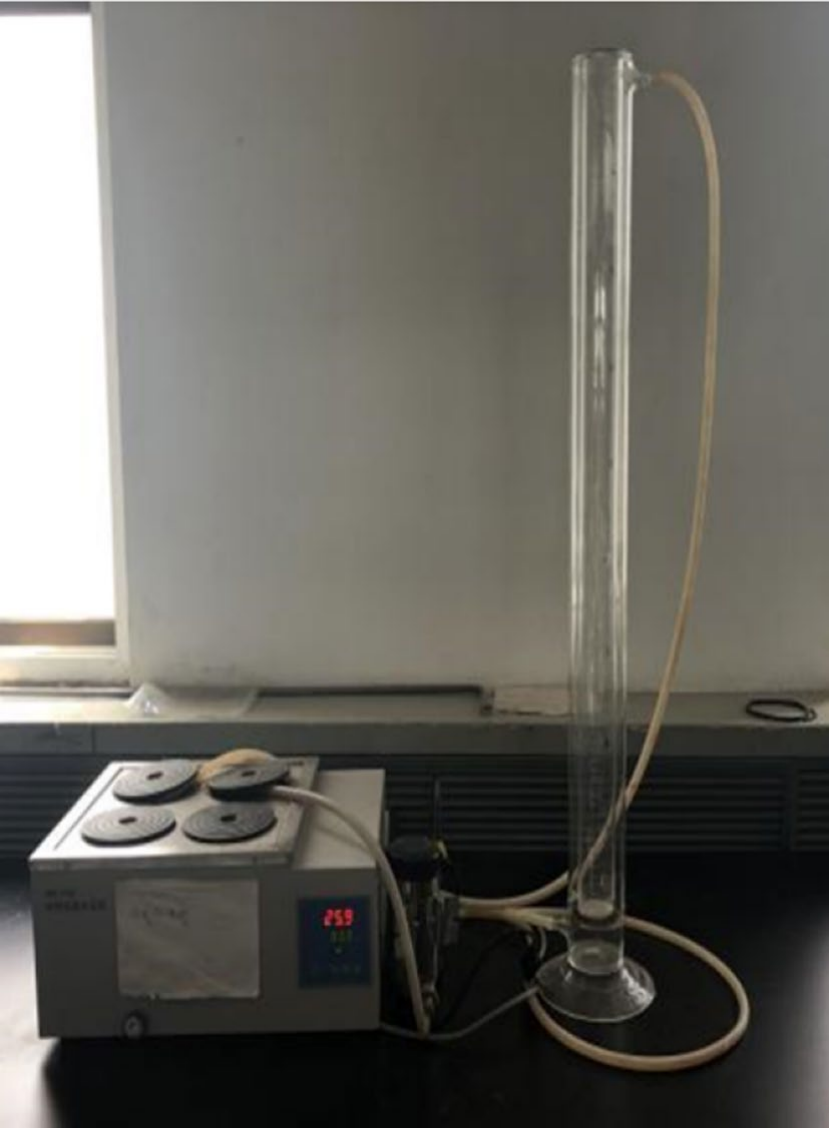
1-L graduated cylinder for measuring the settling rate of proppants with a temperature controller.

**Figure 7:**
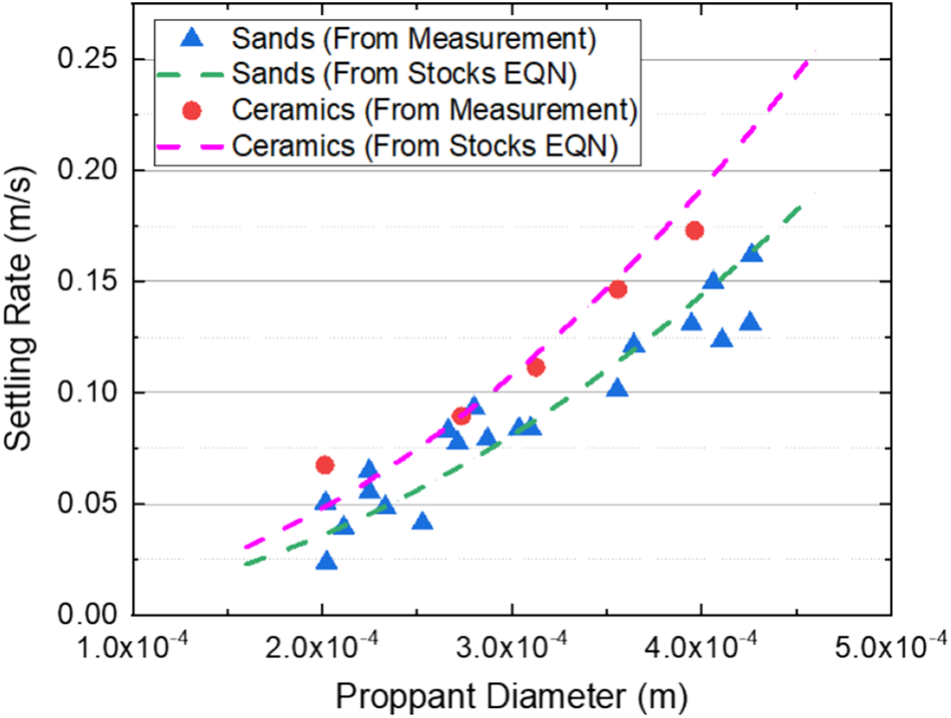
Measured and calculated settling rates of ceramic proppants and silica sands at different diameters.

The settling rate of proppant is measured using a 1-L graduated cylinder with a temperature controller as shown in Fig. 6. The cylinder has an inner diameter of 50 mm, which can minimize the collision effect from the cylinder wall when a micron-sized proppant is settling therein. During the measurement, the cylinder is firstly filled with water; then, water is continuously flowing between the inner wall and outer wall of the cylinder to keep the temperature at 25℃; after the temperature is stabilized, a proppant with a pre-determined diameter is gently placed in the water inside the cylinder. Once the proppant falls to the half-height of the cylinder and its settling rate becomes almost stable, the settling rate is calculated using the settling distance divided by the recorded time. Figure 7 shows the measured settling rates of ceramic proppants and silica sands with different diameters in water, and their settling rates calculated from the Stocks equation. As forecasted by the equation, the settling rate increases with the proppant diameter and decreases with the proppant density.

### Field experiments with different proppants

**Figure 8:**
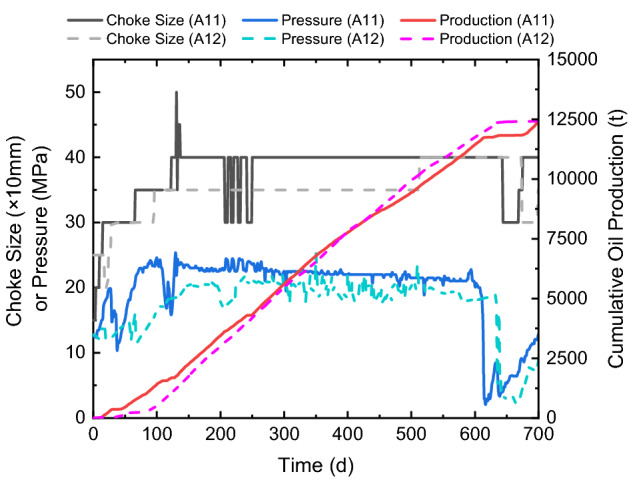
Production curves of comparison wells in the Ma-131 play.

In the Ma-131 play where the reservoir depth is shallower than 3300 m, using silica sands to completely replace ceramic proppants is tested in 14 horizontal wells, and then compared with 17 neighboring wells using ceramic proppants. As can be seen from the conductivity measurement in the lab, the silica sand has a relatively low conductivity value comparing to the ceramic proppant; therefore, when fracturing with silica sands in the field experiments, the proppant volume is increased by 20% to compensate for the conductivity loss compared to the ceramic proppants (i.e., 1.1 times the proppant volume of the ceramic well). Table 1 shows the fracturing parameters of a pair of neighboring wells for testing the performance of different types of proppants. A12 is the control well with ceramic proppants, while A11 is the experimental well with 1.1 times the total proppant volume of A12. Both wells have similar lengths, cluster spacings, and numbers of stages and clusters. Since similar pumping schedules are applied, the water volume used per length increases with the proppant volume used per length. Figure 8 shows the production histories of both wells, where solid curves show the choke size, pressure, and accumulative oil production of A11, while dot curves shows those of A12. Both wells are produced with similar manners as can be seen from the changes of choke size. The pressures of both wells gently decay from 25 to about 20 MPa in 600 days, during which the accumulative oil productions are both about 12,000 t. After 600 days, pressures of both wells drop due to the aggressive flowback of neighboring wells, thus their choke sizes are reduced until neighboring wells are changed back to normal; later, pressures and productions of both wells gradually recover as shown in Fig. 8. In short, no obvious change is noticed when the type of proppants is changed in this pair of neighboring wells.

**Table 1:** Information of comparison wells in the Ma-131 play.

Well name	A11 (1.1 × sands)	A12 (1 × ceramics)
Well length (m)	1605	1604
Stage number	21	22
Cluster number	41	42
Cluster spacing (m)	39.1	38.2
Proppant volume per length (m^3^/m)	1.13	1.00
Water volume per length (m^3^/m)	16.1	15.3
Proppant volume per cluster (m^3^)	44.2	38.2
Water volume per cluster (m^3^)	630.3	584.3

**Figure 9:**
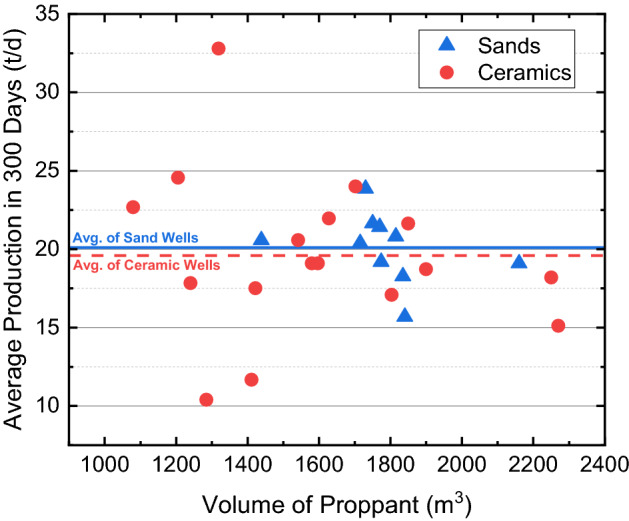
Cross plot of proppant volumes and well productions of 31 wells in the Ma-131 play.

Figure 9 shows the average daily production rates of all 31 wells in the Ma-131 play for the proppant-comparison experiments. Silica sands are used in 14 wells, whose production ranges from 15 to 25 t/d, with an average of 20.1 t/d; ceramic proppants are used in the rest 17 wells, whose average production is 19.6 t/d. In the reservoir with a depth of less than 3300 m, the effective closure pressure is smaller than 30 MPa, and no obvious difference is noticed when ceramic proppants are replaced by silica sands during hydraulic fracturing.

The Ma-18 play has a depth larger than 3500 m, and the horizontal stress can be as large as 75 MPa. During the production where the pore pressure decays with time, the effective closure pressure can increase from 40 to almost 60 MPa. Therefore, silica sands cannot completely replace ceramic proppants in field experiments; 50% sands are mixed with 50% ceramics, while the total proppant volume is 1.1 times that of the pure ceramic wells. Table 2 shows the fracturing parameters of a pair of neighboring well for the experiment in the Ma-18 play. B03 is the control well with ceramic proppants, while B05 is the experimental well using the mixture of silica sands and ceramic proppants. Both wells have similar lengths, cluster spacings, and numbers of stages and clusters. Similar to the well pair shown in Table 1, the water volume increases with the proppant volume, and B05 also has 1.1 times the total water volume of B03 because of the similar pumping schedules.

**Table 2:** Information of comparison wells in the Ma-18 play.

Well name	B05 (1.1 × proppants; 50% sands + 50% ceramics)	B03 (1 × ceramics; 100% ceramics)
Well length (m)	1144	1550
Stage number	16	22
Cluster number	31	43
Cluster spacing (m)	37.6	35.7
Proppant volume per length (m^3^/m)	1.07	0.97
Water volume per length (m^3^/m)	17.0	16.2
Proppant volume per cluster (m^3^)	39.5	34.9
Water volume per cluster (m^3^)	627.4	584.0

**Figure 10:**
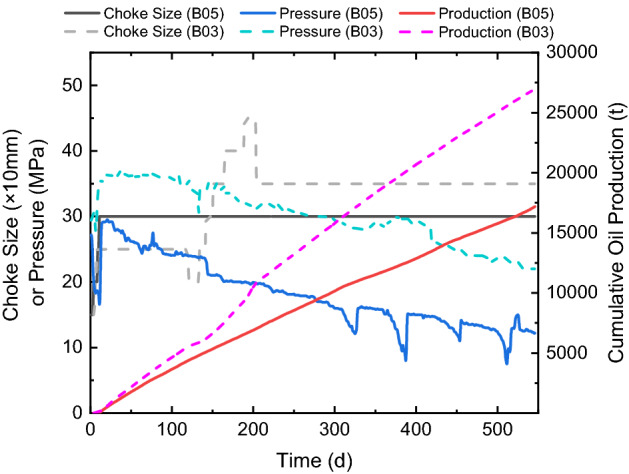
Production curves of comparison wells in the Ma-18 play.

**Figure 11:**
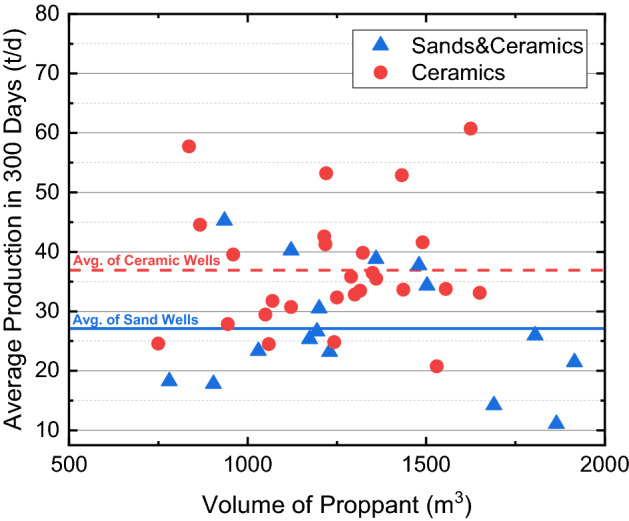
Cross plot of proppant volumes and well productions of 43 wells in the Ma-18 play.

Figure 10 shows the production histories of both wells, where solid curves show the choke size, pressure, and accumulative oil production of B05, while dot curves shows those of B03. When 50% sands are used, both the pressure and cumulative oil production reduce to 2/3 of those of the neighboring well using 100% ceramic proppants. Figure 11 further shows the average daily production rates of all 43 wells in the Ma-18 play for the proppant-comparison experiments. Mixtures of silica sands and ceramic proppants are used in 16 wells, whose production ranges from 10 t/d to 45 t/d, with an average of 27.1 t/d; pure ceramic proppants are used in the rest 27 wells, production ranges from 20 to 60 t/d, with an average of 36.9 t/d. Results indicate that the oil production rate increases since the reservoir pore pressure increases with depth; however, the effective closure stress increases more significantly with depth, which aggravates the crushing of silica sands and the fine migrant that further plugs the propped fracture and reduces the conductivity of the created fracture. In the Ma-18 play where the reservoir depth is larger than 3500 m, silica sands cannot be used as proppants.

In summary, laboratory measurements have shown that the settling rate of the ceramic proppant is 1–1.5 times larger than the silica sand, which results in a less migration rate of the proppant-bank within the created fracture; meanwhile, the conductivity of the propped fracture by ceramic proppants is 2–5 times of that by silica sands at closure pressure less than 55 MPa. However, due to the limitation of laboratory measurement, proppant migration in the fracture and the conductivity of the propped fracture cannot be evaluated at the same time; secondly, although reservoir rock samples are used in the conductivity measurement as shown in “Laboratory measurements of proppant conductivity and settling rate” section, the mimicked fracture faces are flat that ignores the impact of face roughness on proppant migration and fracture conductivity; thirdly, simulation studies have also shown that the closure pressure can affect the flow pattern of fluid within the fracture with rough fracture faces and thus the fracture conductivity^31^. Therefore, field experiments are crucial to evaluating the performance of different proppants that can consider the impact factors ignored in the lab. In reservoirs shallower than 3300 m, wells with pure silica sands perform similarly to ones with pure ceramic proppants, which indicates that the production loss due to the reduced fracture conductivity can be compensated by the increased migration rate of proppants when the cheaper silica sands are applied; therefore, the silica sands are promising in these shallow reservoirs in future development. In reservoirs deeper than 3500 m, oil production can be decreased by 30% when 50% ceramic proppants are replaced by silica sands; this agrees with the results of conductivity measurements and further confirms that conductivity is the controlling factor in these deep reservoirs. A faster migration rate of silica sands meanwhile leads to a narrower width of the propped fracture, which is more susceptible to the closure pressure. Therefore, high-strength proppants are still the best option for deep reservoirs in Mahu.

### Field experiments with different fracture spacing

Decreasing the fracture spacing can aggravate the stress shadow effect and increase the closure stress that affects the conductivity of silica sands. Besides, when the “fracture swarm” forms, the fracture spacing can be significantly smaller than the designed one^32^. Therefore, the impact of fracturing spacing is evaluated for wells using silica sands that are sensitive to the change of stress in the Ma-131 play. Table 3 shows the fracturing parameters of four neighboring wells for evaluating the impact of fracture spacing on the conductivity of sand-propped fractures. A13 and A14 are control wells with the normal design as ones shown in Table 1, while A15 and A16 are experimental wells with smaller fracture spacings. The cluster number of each well is doubled within one fracturing stage, but volumes of proppants and water per stage still remain the same to keep the investment per well almost unchanged. As can be seen from this table, the proppant volume per length are all about 1.2 m^3^/m and the water volume per length are all about 20 m^3^/m; however, the total proppant volume per cluster decreases from 40 to 20 m^3^/m, and the total water volume per cluster decreases from 600 to 350 m^3^/m as the cluster per stage increases from 2 to 3 in the experimental wells.

**Table 3:** Information of comparison wells with different fracture spacings.

Well name	A13 (normal design)	A14 (normal design)	A15 (smaller spacing)	A16 (smaller spacing)
Well length (m)	1554	1427	1530	1530
Stage number	22	18	36	29
Cluster number	50	43	104	80
Cluster spacing (m)	30.3	27.8	15.1	18.9
Proppant volume per length (m^3^/m)	1.16	1.29	1.19	1.20
Water volume per length (m^3^/m)	17.6	19.0	20.4	21.8
Proppant volume per cluster (m^3^)	36.1	42.8	17.5	23.0
Water volume per cluster (m^3^)	547.0	630.5	300.1	416.9

**Figure 12:**
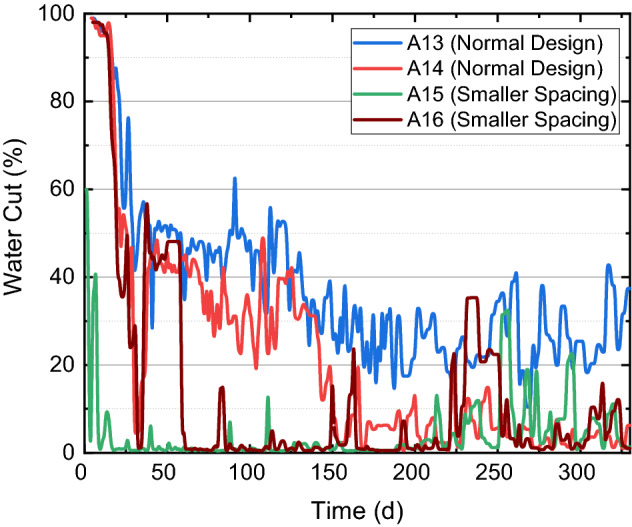
Change of water cut of comparison wells with different fracture spacings.

**Figure 13:**
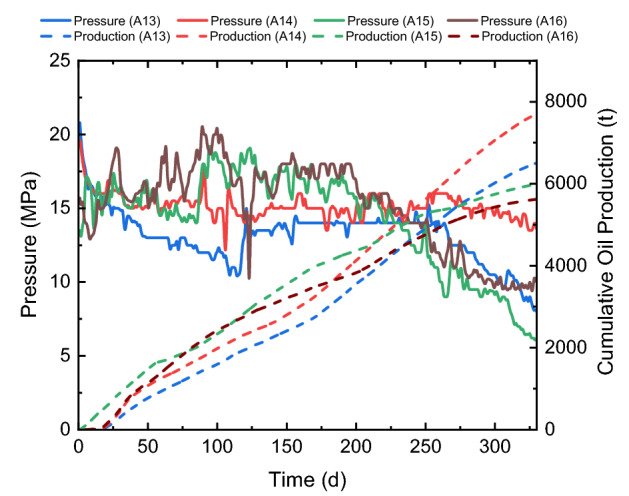
Production curves of comparison wells with different fracture spacings.

Figure 12 shows changes of the water cut of four wells. When the fracture spacing decreases and more fractures are designed within one fracturing stage, the fracturing fluid per cluster decreases, and the leak-off of fracturing fluid into the reservoir rock increases. These result in smaller water cut and thus a smaller flowback ratio during the production in A15 and A16. Figure 13 shows the production histories of four wells, where solid curves show the pressure and dot curves show the cumulative oil production of them. Compared with A13 and A14, A15 and A 16 have relatively high productions in the first 200 days, but their pressures decay faster which makes their productions lower than wells with normal designs after one year of production. When the fracture spacing decreases, the reservoir rock is better exposed to the created fracture network, which contributes to the production enhancement at the early time of production. However, since the proppant volume is kept constant per unit length, created fractures cannot be well propped when the fracture spacing decreases; furthermore, the decrease of fracture spacing also increases the closure stress, especially when the pore pressure decays with oil production, which aggravates the crushing of silica sands and the decrease of fracture conductivity. This group of field experiments indicates that sufficient sands are necessary for each fracture (at least 30 m^3^) to obtain an economic production rate when the fracture spacing decreases to 15 m or smaller in the Ma-131 play.

### Field experiments with different fracturing fluids

The crosslinked guar is 3 times the price of the slickwater, and its residuals can cover the fracture face and plug the propped fractures that reduces the well productivity. It has been widely accepted that the crosslinked guar has a good proppant-carrying ability, and thus can generate long and narrow propped fractures. When the slickwater is used, a large pumping rate is needed to compensate the loss of proppant-carrying ability; it likely generates short and wide propped fractures (especially in the near-wellbore region), and can create a complex fracture network in weakly-consolidated reservoirs or reservoirs with natural fractures. The Mahu reservoir has a relatively large permeability that requires the propped fractures to have a large conductivity to match; meanwhile, the reservoir with a thickness of a few tens of meters has little demand for vertical supporting of proppants. Therefore, slickwater can be a promising option, and thus it is evaluated through field experiments.

**Figure 14:**
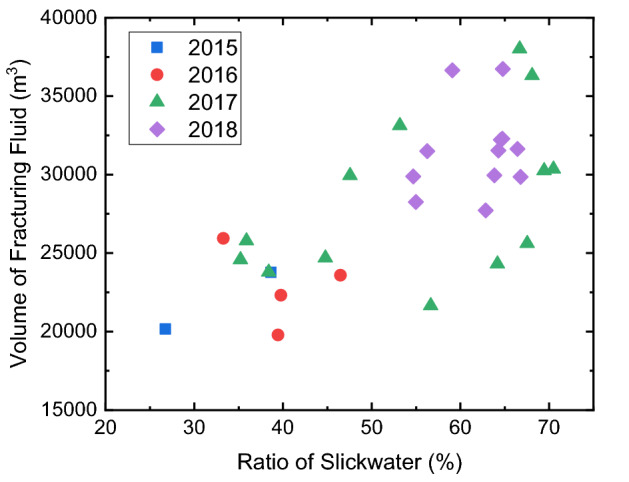
Cross plot of slickwater ratio and total fracturing fluid volume of experimental wells in 4 years.

**Figure 15:**
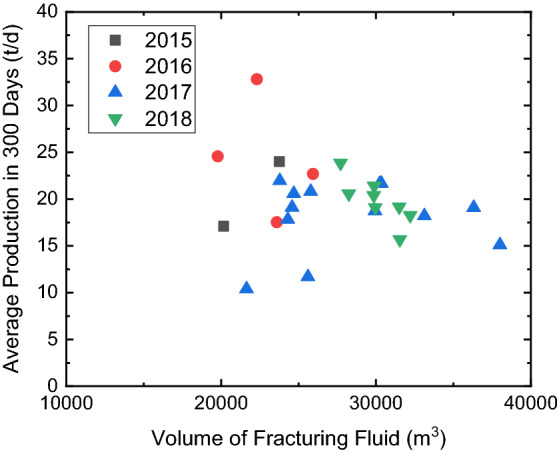
Cross plot of total fracturing fluid volume and average annual production of experimental wells in 4 years.

From 2015 to 2018, the slickwater ratio is gradually increased, and it is expected to evaluate if slickwater can completely replace the crosslinked guar in the Mahu reservoir. Figure 14 shows the cross plot of the slickwater ratio and the total volume of fracturing fluid of 31 experimental wells in these 4 Years. Since the friction reducer added in the slickwater can reduce more than 70% of friction pressure of fluid flowing inside the wellbore, a large pumping rate can be applied during hydraulic fracture. Therefore, when the ratio of slickwater increases, the total volume of the fracturing fluid used per well also increases, and the intensity of fracturing fluid can reach 20 m^3^/m. Figure 15 shows the cross plot of the total volume of fracturing fluid and the average annual production of experimental wells in these 4 years. Although the volume of fracture fluid is significantly increased, no obvious enhancement of the oil production is observed. Since the volume of proppants per cluster remains the same, the potential increase of fracture volume does not lead to an increase of the propped fracture volume. When the slickwater ratio increases, a large pumping rate can prevent the proppant plugging during the operation, but no production enhancement should be expected without increasing the proppant volume per cluster.

**Figure 16:**
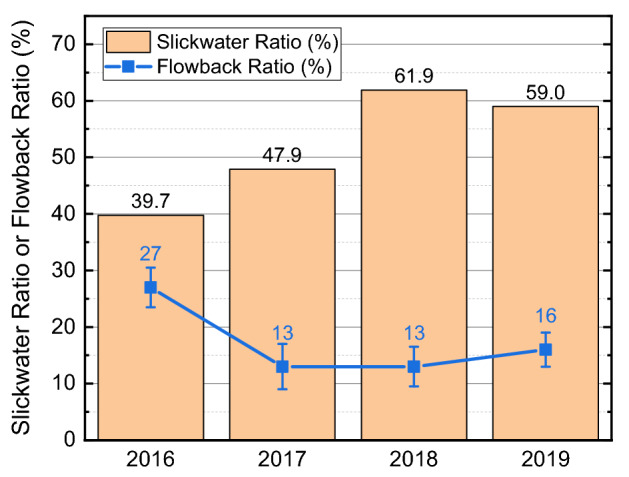
Change of average slickwater ratio and average flowback ratio in 4 years.

Figure 16 shows the change of average slickwater ratio and flowback ratio in 4 years. Compared with the crosslinked guar, slickwater is significantly less viscous and has a large leak-off coefficient. When the fracturing fluid leaks off into the reservoir rock, the created fracture can close and be well propped at the closure pressure. This can potentially prevent the flowback of proppant and retain the conductivity of the propped fracture during oil production. In all, reducing the usage of the crosslinked guar and increasing the slickwater ratio can reduce the operation cost without obviously affecting the migration of proppants and the well productivity. Therefore, replacing the crosslinked guar with slickwater is recommended for future fracturing designs.

## Conclusion

Field experiments have been conducted since 2017 to explore economical and effective fracturing parameters for developing the Mahu tight oil reservoirs. This work summarizes and analyzes observations from experimental wells where different types of proppants and fracturing fluids, slickwater ratios, and fracture spacings are tested in the Ma-131 and Ma-18 plays.

Different types of silica sands and ceramic proppants are first evaluated in the lab, where the reservoir rock is cored and processed for the conductivity measurement. Results indicates that embedment is not obvious even for the high-strength ceramic proppants, and the crushing is the main reason causing the conductivity reduction of propped fractures. For widely used 40/70 mesh proppants, silica sands losses conductivity above an effective closure pressure of 40 MPa, while ceramic proppants can retain a conductivity of 3.5 D cm at 69 MPa. This agrees well with results from the field experiments, where 100% silica sands have similar performance as 100% ceramic proppants in reservoirs shallower than 3300 m, but 50% ceramic proppants with 50% silica sands can only reach 70% of production rates of wells with 100% ceramic proppants in reservoirs deeper than 3500 m. In shallower reservoirs where silica sands still have conductivity above 1 D cm, the reduction of fracture conductivity can be compensated by the increase of migration rate of proppant-bank; in deeper reservoirs, the faster migration rate of silica sands leads to the narrower width of the propped fracture, which becomes more susceptible to the closure pressure.

When silica sands are applied, decreasing the fracture spacing increases the closure stress that aggravates sand crushing and thus decreases the fracture conductivity. In such cases, increasing the cluster number within a stage is not recommended unless the sand volume can be kept above 30 m^3^ per cluster. Besides, results from field experiments also indicate that increasing the slickwater ratio can reduce the fracturing cost without sacrificing the oil production rate in the Ma-131 play. This is because the slickwater can significantly reduce the formation damage; meanwhile, it allows increasing the pumping rate during fracturing and can increase the stimulation volume, which compensates the potential loss of fracture conductivity due to its poor sand-carrying ability.
